# Supplementary material for: Deep Phenotyping of Superficial Epidermolytic Ichthyosis due to a Recurrent Mutation in KRT2
Source: Int J Mol Sci. 2022 Jul 14;23(14):7791. doi: 10.3390/ijms23147791 (PMC9317500; doi:10.3390/ijms23147791)
Supplement: Supplementary file 1 [file ijms-23-07791-s001.zip › ijms-1797713-supplementary.pdf]

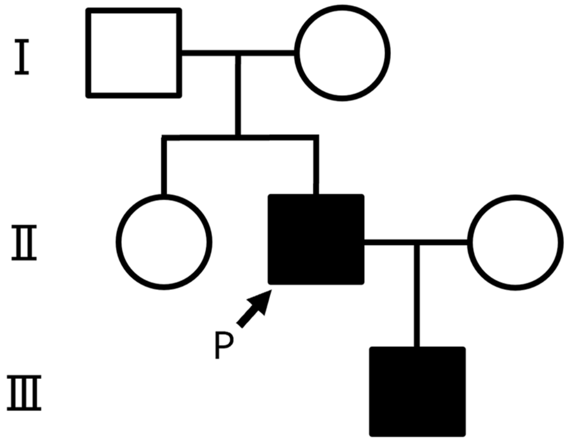

Figure S1. The pedigree of the present family. Squares are males and circles females. Solid squares represent affected individuals (P, the proband).

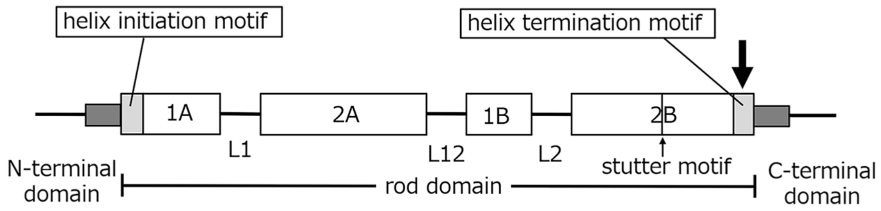

Figure S2. The domain structure of keratin 2 and the mutation detected in the present patient. The location of the mutation in this patient is indicated by the black arrow.
